# The Effect of a Fast-Releasing Hydrogen Sulfide Donor on Vascularization of Subcutaneous Scaffolds in Immunocompetent and Immunocompromised Mice

**DOI:** 10.3390/biom10050722

**Published:** 2020-05-06

**Authors:** Alexandra M. Smink, Avid Najdahmadi, Michael Alexander, Shiri Li, Samuel Rodriquez, Harry van Goor, Jan-Luuk Hillebrands, Elliot Botvinick, Jonathan R. T. Lakey, Paul de Vos

**Affiliations:** 1 Department of Pathology and Medical Biology, University Medical Center Groningen, University of Groningen, 9713 GZ Groningen, The Netherlands; h.van.goor@umcg.nl (H.v.G.); j.l.hillebrands@umcg.nl (J.-L.H.); p.de.vos@umcg.nl (P.d.V.); 2 Department of Surgery, University of California Irvine, Orange, CA 92868, USA; michaela@hs.uci.edu (M.A.); shiril@uci.edu (S.L.); samuemr1@uci.edu (S.R.); elliot.botvinick@uci.edu (E.B.); jlakey@uci.edu (J.R.T.L.); 3 Department of Chemical Engineering and Materials Science, University of California Irvine, Irvine, CA 92617, USA; avid.najd@gmail.com; 4 Department of Biomedical Engineering, University of California Irvine, Irvine, CA 92617, USA

**Keywords:** immune system, vascularization, hydrogen sulfide, subcutaneous scaffolds

## Abstract

Islet transplantation into subcutaneous polymer scaffolds has shown to successfully induce normoglycemia in type 1 diabetes models. Vascularization of these scaffolds is imperative for optimal control of glucose levels. We studied the effect of the vascular stimulator hydrogen sulfide (H_2_S) on the degree of vascularization of a scaffold and the role of the immune system in this process. Scaffolds were subcutaneously implanted in immunocompetent C57BL/6 and immunocompromised nude mice. Mice received twice-daily intraperitoneal injections of the fast-releasing H_2_S donor sodium hydrosulfide (NaHS, 25 or 50 μmol/kg) or saline for 28 days. After 63 days the vascular network was analyzed by histology and gene expression. Here we showed that the vascularization of a subcutaneous scaffold in nude mice was significantly impaired by H_2_S treatment. Both the CD31 gene and protein expression were reduced in these scaffolds compared to the saline-treated controls. In C57BL/6 mice, the opposite was found, the vascularization of the scaffold was significantly increased by H_2_S. The mRNA expression of the angiogenesis marker CD105 was significantly increased compared to the controls as well as the number of CD31 positive blood vessels. In conclusion, the immune system plays an important role in the H_2_S mediated effect on vascularization of subcutaneous scaffolds.

## 1. Introduction

Transplantation of insulin-producing cells, such as the islets of Langerhans, is a promising treatment for type 1 diabetes. It provides continuous regulation of the blood glucose levels and therefore results in stable glycemic control and the reduction of secondary complications such as cardiovascular diseases, retinopathy, and nephropathy. Currently, pancreatic islets are infused via the portal vein into the liver and this results in 100% insulin independency after 1 year [1], but due to multiple factors, the islets do not survive long-term since insulin independency decreases to less than 50% after 5 years. There are many indications that the liver is partly responsible for this and therefore may not be the optimal islet transplantation site [2]. The microenvironment of the liver is detrimental for islet survival due to the presence of specific liver immune cells, the induced blood-mediated inflammatory response, and the exposure to antigens and toxins because of the gut-liver axis [2]. 

Since the human body does not provide a better islet transplantation site, engineering an artificial islet transplantation site, also called a scaffold, that mimics the pancreatic microenvironment, could be a solution [3]. Such a scaffold could be implanted under the skin, making transplantation of insulin-producing cells only a minor surgical procedure and allowing for retrieval of these cells. Retrievability might be mandatory with replenishable cell sources, like stem cells, that still have some insufficiencies such as incomplete maturation, uncontrolled proliferation, aberrant protein expression, and tumorigenic potential [4].

An artificial islet transplantation site with proven success is the subcutaneously implanted poly (D,L-lactide-co-ε-caprolactone) (PDLLCL) scaffold [5,6]. After transplantation of 800 islets into this scaffold, 80% of the mice became normoglycemic, whereas islet transplantation under the unmodified skin without the scaffold did not result in normoglycemia in any of the mice. Crucial for islet function is their vascularization. This is not only important for the supply of oxygen and nutrients, but also for their ability to quickly respond to glucose changes in the blood. In the native pancreas, islets receive 10–20% of the pancreatic blood supply, while the total islet mass is just 2% [7]. Furthermore, the number of capillaries in the islets is ten times higher than in the rest of the pancreas [7], and these capillaries are 20–30% wider and form a glomerular-like network around the insulin-producing cells [8]. However, during islet isolation from donor pancreata, the islet vasculature is disrupted. Therefore, improving the vascularization of islet transplantation scaffolds would further increase transplantation outcomes. 

The gasotransmitter hydrogen sulfide (H_2_S) has been reported to be involved in the homeostasis of the cardiovascular system [9]. In vitro H_2_S stimulates endothelial cell proliferation, migration, and tube formation [10,11]. In vivo it promotes blood vessel sprouting and growth in chicken chorioallantoic membranes [11] and a study in mice showed increased angiogenesis in subcutaneous Matrigel plugs [10]. Therefore, we aimed to improve the vascularization of our polymer scaffold with the use of H_2_S. Since H_2_S is also known to influence the immune system [9], we studied the scaffold vascularization in both immunocompromised and immunocompetent mice. Vascularization was investigated by performing immunohistochemistry and gene expression analysis of several vascular markers. This study will support determination of the optimal preimplantation period needed for the development of a vascular network before introduction of islets. 

## 2. Materials and Methods

### 2.1. Experimental Design

Immunocompromised athymic nude mice (Foxn1nu) and immunocompetent C57BL/6 mice were used to test the effect of H_2_S on vascularization of PDLLCL scaffolds. Scaffolds were subcutaneously implanted on the back of the mice. From the day of implantation, the mice received an intraperitoneal injection of the fast-releasing H_2_S donor sodium hydrosulfide (NaHS) twice daily until one month after implantation. One group of mice (*n* = 6) received a low dose of NaHS (25 μmol/kg), the other group (*n* = 6) received a high dose of NaHS (50 μmol/kg), and mice receiving saline injections served as a control group (*n* = 6). To allow the vascular network to stabilize, the scaffolds were not directly removed after finishing the H_2_S treatment period. Non-invasive, in vivo oxygen measurements in the nude mice showed no statistical differences between the groups and it seemed to stabilize after 63 days (data not shown). Therefore, scaffolds were removed for histological analysis and quantification of vascularization by real time reverse transcription polymerase chain reaction (RT-PCR) after 63 days.

### 2.2. Scaffold Preparation

Scaffolds were prepared from a 4% (w/v) PDLLCL solution in chloroform (Sigma-Aldrich, Zwijndrecht, The Netherlands). The PDLLCL solution was thoroughly mixed with sodium chloride particles (Sigma-Aldrich) of 250–425 μm (10:1 w/w) in order to create a porous structure. This solution was transferred into sterile glass petri dishes to allow the solvent to evaporate. To remove the salt particles, the polymer sheet (5 mm thick) was extensively washed with sterile H_2_O. The polymer sheet was casted and resized, resulting in 10 mm by 15 mm devices. The scaffolds were stored in 70% ethanol for several days to sterilize them before implantation. Fibrin was added to the scaffold on the day of implantation. Briefly, a 2 mg/mL fibrinogen solution (Sigma-Aldrich) was mixed with 100 U/mL thrombin IIa (Sigma-Aldrich) (100:1) and transferred into the pores of the scaffolds. 

### 2.3. Animals

The University of California Institutional Animal Care and Use Committee at the University of Irvine approved all described animal procedures (IACUC # 2008-2850). Scaffolds were implanted in 8-week old male athymic nude mice (total of 18 mice; Charles River, Wilmington, NC, USA) and C57BL/6 mice (total of 18 mice; Charles River). Animals were housed at the University of California Irvine animal facility and maintained under 12-hour light/dark cycles with ad libitum access to water and standard chow.

### 2.4. Implantations and NaHS Injections

Mice were anesthetized by placing them individually in an induction chamber infused with 4% isoflurane (Patterson Veterinary, Greeley, CO, USA) mixed with 100% oxygen (flow rate 2 L/min). After induction, the mice were placed on a homeothermic blanket with an anesthesia facemask and the anesthesia was reduced during surgery to 2%. A small incision was made in the skin, after which a subcutaneous pocket was created on the back of the mice to implant the PDLLCL scaffolds. The skin was closed using skin staples (Cellpoint Scientific, Gaithersburg, MD, USA). All mice received ibuprofen water (Banner Pharmacaps, High Point, NC, USA; 0.2 mg/mL) as an analgesic post-surgery for two days. Since H_2_S is a gas, a NaHS solution (Sigma-Aldrich) was used as a H_2_S donor. The solution was prepared shortly before injection by dissolving NaHS in saline. From the day of implantation, the mice received an intraperitoneal injection of NaHS or saline twice daily until 28 days after implantation of the scaffolds. The injected volume, based on the dose and body weight, ranged between 250–350 μL for the H_2_S groups. Control mice received a similar volume of saline.

### 2.5. Histology 

The scaffolds were removed 63 days after implantation. Half of the scaffold was processed for histology (*n* = 6 of each experimental group) and the other half was used for RT-PCR (*n* = 6 of each experimental group; described below). For histology, scaffolds were fixed in 2% paraformaldehyde and processed for paraffin embedding. Paraffin sections were stained for the endothelial marker CD31 (1:200; R&D Systems, Abingdon, UK) and the monocyte and macrophage marker MOMA-2 (1:50; Abcam). Briefly, for the CD31 staining, antigen retrieval was performed by incubation in 10 mM citrate buffer (pH 6.0) for 15 min at 100 °C. This was followed by blocking with 5% donkey serum (Sigma-Aldrich) and an overnight incubation with the primary antibody. The secondary donkey anti-goat alkaline phosphatase conjugated antibody (1:100; Abcam) was applied for 45 min. For the MOMA-2 staining, a 15 min incubation with 0.1% trypsin (in 6.8 mM 0.1% calcium chloride and 0.1 M Tris(hydroxymethyl)-aminomethane-hydrogen chloride, pH 7.8) at 37 °C was performed for antigen retrieval, followed by a blocking step with 5% rabbit serum (Agilent Technologies). After overnight incubation with the primary antibody, a biotin conjugated rabbit anti-rat antibody (1:100; Vector Laboratories, Peterborough, UK) was added for 45 min. Subsequently, sections were incubated for 45 min with a streptavidin-alkaline phosphatase conjugate (1:100; Southern Biotech). Alkaline phosphatase activity of both above-mentioned stainings was demonstrated by incubating the sections for 10 min with SIGMAFAST^TM^ Fast Red. A short incubation with hematoxylin was used as counterstain. 

All stained sections were scanned with a Hamamatsu Nanozoomer slide scanner (Hamamatsu, Almere, The Netherlands). Scans were analyzed using Aperio ImageScope software (Leica Microsystems B.V., Rijswijk, The Netherlands). The number of CD31 positive structures were counted and corrected for the measured scaffold area (mm^2^). For the MOMA-2 staining, the number of strong positive pixels was corrected for the measured scaffold area (mm^2^). The fold change of both stainings was calculated relative to the control. Pictures were taken using a Leica DM 2000 LED microscope with a Leica DFC 450 camera (Leica Microsystems B.V.). 

### 2.6. RT-PCR

To quantify gene expression in the scaffolds, half of each individual scaffold at day 63 was processed for RT-PCR. Briefly, total RNA was isolated using Trizol according to manufacturer’s protocol (Invitrogen; Thermo Scientific, Landsmeer, The Netherlands). The RNA concentration was determined using a NanoDrop 1000 spectrophotometer (NanoDrop products, Wilmington, NC, USA). cDNA was reverse transcribed using a SuperScript^®^ III Reverse Transcriptase kit according to the instructions of the manufacturer (Life technologies). RT-PCR was conducted using ViiATM Real Time PCR system (Life technologies) with primer and probe sets (TaqMan Gene Expression Assays) for several vascularization genes (CD31, VEGFa, VE-cadherin, CD105 (endoglin), angiopoietin 1 and 2) purchased from Thermo Scientific (Table 1) and qPCR Mastermix Plus (Eurogentec, Seraing, Belgium). Reactions were performed at 50 °C for 2 min, 95 °C for 10 min, 95 °C for 15 s, and 60 °C for 60 s, repeating these last two steps for 40 cycles. Delta Ct values were calculated and normalized against the expression of the housekeeping gene GAPDH. Delta Ct values were used to determine the fold change of the H_2_S treated groups compared to the control group. 

### 2.7. Blood Vessel Functionality Tests

To test the functionality of the blood vessels, 3 out of the total of 6 mice in each C57BL/6 group were used for in vivo oxygen measurements and lectin perfusion. The oxygen measurements were done with the Microx 4 precision sensing system for oxygen profiling from PreSens (Regensburg, Germany) at day 7, 14, 28, and 63 after implantation. To this end, the oxygen probe was placed in a minimal invasive manner in the scaffold for 10 min while the animal was anesthetized with isoflurane (as described above for the implantations). Furthermore, mice were perfused with 200 μL DyLight 649 labeled Lycopersicon Esculentum (Tomato) lectin (Vector Laboratories; Brunschwig Chemie, Amsterdam, The Netherlands) via a single injection into the carotid artery under isoflurane anesthesia (as described above for the implantations) at day 63. Lectin was allowed to circulate for 15 min after which the vasculature was flushed with saline. Explanted scaffolds were fixed in 2% paraformaldehyde and confocal microscopy (Leica SP8; Leica Microsystems B.V.) was used to obtain images of the lectin staining. Acquired images were analyzed with Image J [12] as described by Weaver et al. in 2018 [13]. Briefly, the background fluorescent signal was removed and the number of branches and junctions, the maximal branch length, and the average branch length were measured using the skeleton macro plugin. 

### 2.8. Statistics

Statistical analysis was carried out in GraphPad Prism [14]. A Shapiro–Wilk normality test was performed to test the data for normality. To test differences between the groups, a one-way ANOVA with a post-hoc test was applied, and *p*-values < 0.05 were considered significant. The data are presented in mean ± standard error of mean.

## 3. Results

### 3.1. Vascularization of the Scaffolds in C57BL/6 Mice not Affected by H_2_S

**Figure 1:**
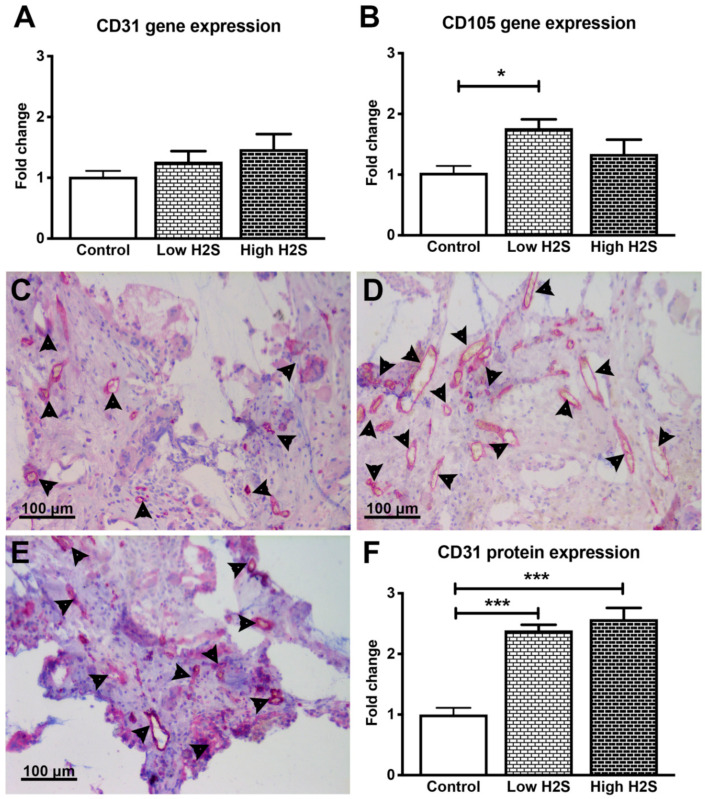
Gene and protein expression of vascularization markers after 63 days of implantation in C57BL/6 mice. There were no significant changes in the mRNA expression of CD31 (**A**), but the expression of CD105 statistically significantly increased after treatment with the low H_2_S dose (**B**; *n* = 6). Many CD31 positive cells (pink color/arrowheads) were found in the control (**C**), low (**D**) and high (**E**) H_2_S treated groups. (**F**) depicts the quantification of the CD31 staining; the number of blood vessels per mm^2^ of scaffold was normalized to the control (*n* = 6). Mean and standard error of the mean are plotted; a statistical analysis was carried out using a one-way ANOVA with a Tukey post-hoc test, *p* < 0.05 (*), *p* < 0.0001 (***).

Since the skin is less vascularized than the native pancreas, vascularization of the subcutaneous implanted scaffold is imperative for transplantation outcomes and efficacy [15]. Previous studies of others showed that H_2_S enhances vascularization [10,11]. Cai and coworkers [10] showed increased neovascularization of a subcutaneous placed Matrigel plug after 7 days of intraperitoneal injections with NaHS (10–50 μmol/kg). This was studied in immunocompetent C57BL/6 mice. Therefore, to determine the effect of H_2_S on vascularization of subcutaneous scaffolds, they were implanted in C57BL/6 mice for 63 days and processed for RT-PCR and histology of vascularization markers. However, H_2_S treatment did not stimulate vascularization at the gene level in the immunocompetent C57BL/6 mice. No significant differences were found between the groups in the gene expression of endothelial marker CD31 (Figure 1A). Furthermore, the same was observed for the gene expression of other vascularization markers such as VE-cadherin, angiopoietin 1, and angiopoietin 2 showing that H_2_S treatment did not significantly increase their expression (Table 2). Only the mRNA expression of angiogenesis marker CD105 was significantly upregulated in the low H_2_S group (fold change 1.8 ± 0.4) compared to the untreated control (Figure 1B). 

At the protein level, both H_2_S dosages significantly increased the number of CD31 positive blood vessels in C57BL/6 mice (Figure 1C–F). The low dose showed an increase of vascularization with a fold change of 2.4 ± 0.1 compared to 1.0 ± 0.1 in the untreated control group. In addition, the fold change of the high dose group was significantly increased (2.6 ± 0.2) compared to the control.

**Figure 2:**
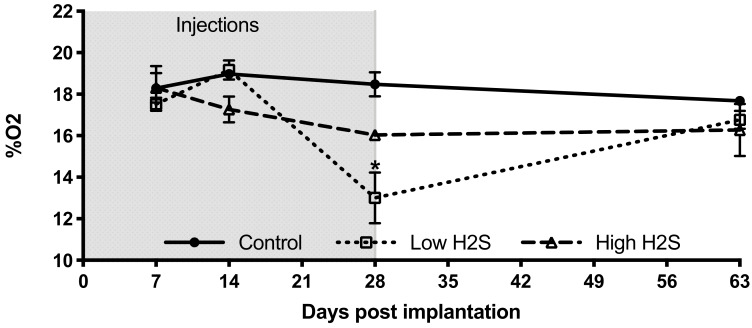
Oxygen percentage within subcutaneous scaffolds of C57BL/6 mice. After implantation of the scaffolds and start of the H_2_S treatment (grey area) the oxygen percentage was measured on day 7, 14, 28, and 63 with the Microx 4 PreSens system. Mean and standard error of the mean are plotted (*n* = 3); a statistical analysis was carried out using a two-way ANOVA with a Bonferroni post-hoc test, *p* < 0.05 (*).

**Figure 3:**
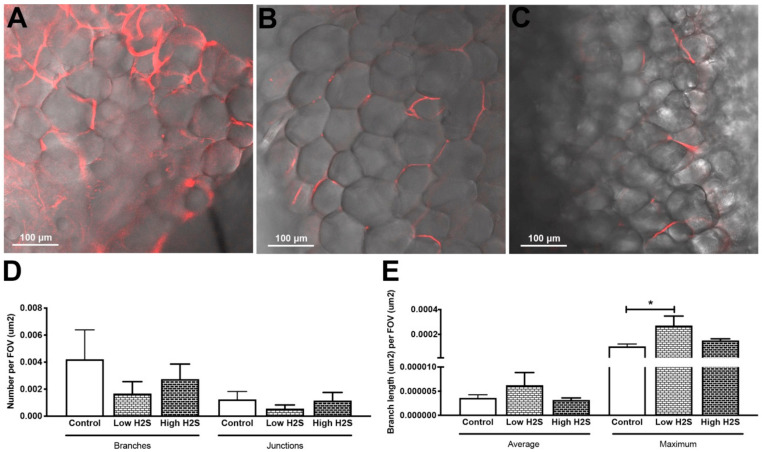
Lectin labeling of blood vessels after 63 days of implantation in C57BL/6 mice and H_2_S treatment. Lectin labeling (red) was found in the control (**A**) group and in the low (**B**), and high (**C**) H_2_S treated groups. (**D**,**E**) depict the quantification of lectin labeling. Mean and standard error of the mean are plotted (*n* = 3); a statistical analysis was carried out using a one-way ANOVA with a Dunnett’s post-hoc test, *p* < 0.05 (*).

To get more insight into the extent that the changes in vascularization by H_2_S improved the available amount of oxygen within the subcutaneous scaffolds, we measured the in vivo oxygen percentage (Figure 2). These measurements showed a statistically significant decrease in oxygen percentage by the low H_2_S treatment at day 28 (*p* < 0.05) compared to both the control and the high H_2_S treatment. The oxygen percentage in the low H_2_S group was 13.0% ± 1.2, whereas the oxygen percentages in the control and high H_2_S groups were respectively 18.5% ± 0.6 and 16.0% ± 0.5. However, this effect was not present any more at day 63. Although we found increased amounts of blood vessels, the amount of available oxygen does not seem to be improved. In addition, the functionality of the blood vessels was further tested by using lectin infusion. The lectin labeling of blood vessels showed that in all groups blood vessels within the scaffolds are well perfused (Figure 3A–C), but no statistical differences were found between the groups in the number of branches or junctions (Figure 3D). In addition, no differences were observed in the average vascular branch length, but the maximal vascular branch length is significantly longer after treatment with the low dose of H_2_S (Figure 3E).

**Figure 4:**
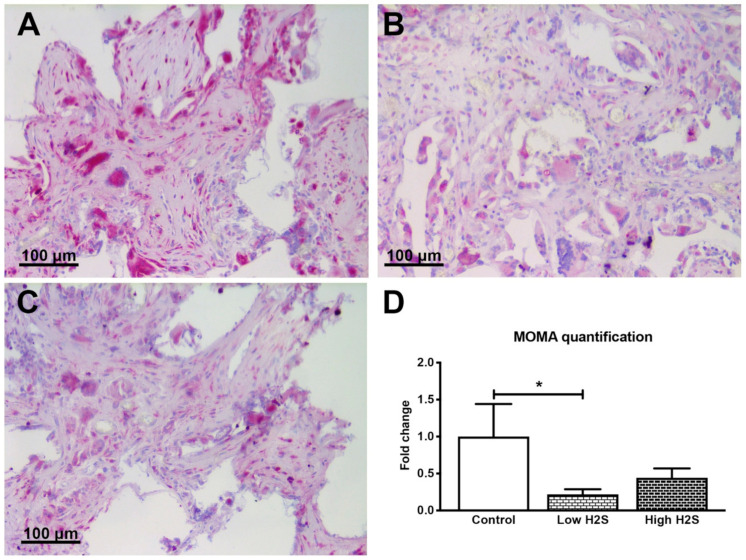
Staining of monocytes and macrophages after 63 days of implantation in C57BL/6 mice. Monocytes and macrophages are stained in pink for the control group (**A**), low dose H_2_S (**B**), and high dose H_2_S (**C**). (**D**) depicts the quantification of the staining; the number of strong positive pixels per mm^2^ of scaffold was normalized to the control (*n* = 6). Mean and standard error of the mean are plotted; a statistical analysis was carried out using a one-way ANOVA with a Dunnett’s post-hoc test, *p* < 0.05 (*).

To gain more insight into the possible mechanisms underlying the vascularization after H_2_S treatment, monocyte and macrophage staining was performed (Figure 4). This showed that monocytes and macrophages were reduced after 63 days of implantation in C57BL/6 mice with H_2_S treatment compared to untreated controls (Figure 4). The fold-change for the low and high H_2_S group was respectively 0.2 ± 0.1 and 0.4 ± 0.2. 

### 3.2. Vascularization of the Scaffolds in Nude Mice Impaired by H_2_S

**Figure 5:**
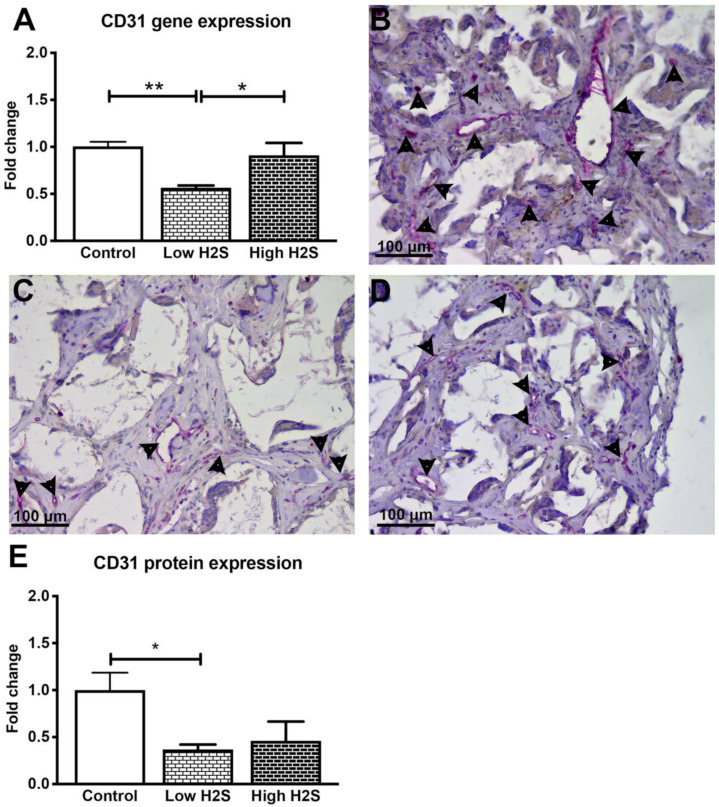
Gene and protein expression of vascularization maker CD31 after 63 days of implantation in nude mice. The expression of CD31 statistically significantly decreased after treatment with the low H_2_S dose (**A**; *n* = 6). Many CD31 positive cells (pink color/arrowheads) were found in the control (**B**) group, the presence of CD31 was decreased in the low (**C**) and high (**D**) H_2_S treated groups. (**E**) depicts the quantification of the CD31 staining; the number of blood vessels per mm^2^ of scaffold was normalized to the control (*n* = 6). Mean and standard error of the mean are plotted; a statistical analysis was carried out using a one-way ANOVA with a Tukey post-hoc test, *p* < 0.05 (*), *p* < 0.001 (**).

The reduced influx of monocytes and macrophages in the C57BL/6 mice in response to H_2_S indicates a role for the immune system. Therefore, to investigate if the immune status of the animal plays a role in the effect of H_2_S, we repeated this study in nude mice. Again after 28 days of treatment and 63 days of implantation, the scaffolds were examined by gene expression analysis and histology. Unlike in the C57BL/6 mice, statistically significant differences were found in the mRNA expression levels of CD31 (Figure 5A). In contrast to our hypothesis, H_2_S did not stimulate vascularization. Compared to the control group, the mRNA expression of the endothelial marker CD31 was significantly reduced by 50% when treated with a low dose of H_2_S (fold change of 0.6 ± 0.02, *p* < 0.001). Treatment with the high H_2_S dose did not result in a significant decrease (fold change of 0.9 ± 0.1). Furthermore, the gene expression of other vascularization markers such as VEGFa and VE-cadherin decreased in a dose-dependent manner with H_2_S treatment, whereas the expression of angiopoietin 1 increased in a dose-dependent manner. The expression of angiopoietin 2 and the angiogenesis marker CD105 remained unchanged (Table 3). This is in contrary with the results from the C57BL/6 mice, where only CD105 was found to significantly increase after treatment with H_2_S.

Immunohistological staining of CD31 showed a similar trend in the protein level (Figure 5B–E) as was observed for mRNA expression. The treatment with H_2_S resulted in a decreased number of blood vessels, whereas the C57BL/6 mice showed a significant increase of blood vessels after H_2_S treatment. Treatment with the low dose resulted in a statistically significant decrease (*p* < 0.05) of CD31 positive structures with a fold change of 0.4 ± 0.1. The high dose also showed a decrease, the number of blood vessels was halved in these scaffolds (fold change 0.5 ± 0.2) compared to the controls (*p* = 0.07). We observed in the raw data of the control groups a higher number of CD31 positive blood vessels per mm^2^ of scaffold, 40.5 ± 7.5 and 11.0 ± 1.2, in the nude mice compared to the C57BL/6 mice, respectively (Figure S1A). In addition, H_2_S did not influence the in vivo oxygen availability within the scaffolds in the nude mice (Figure S2).

**Figure 6:**
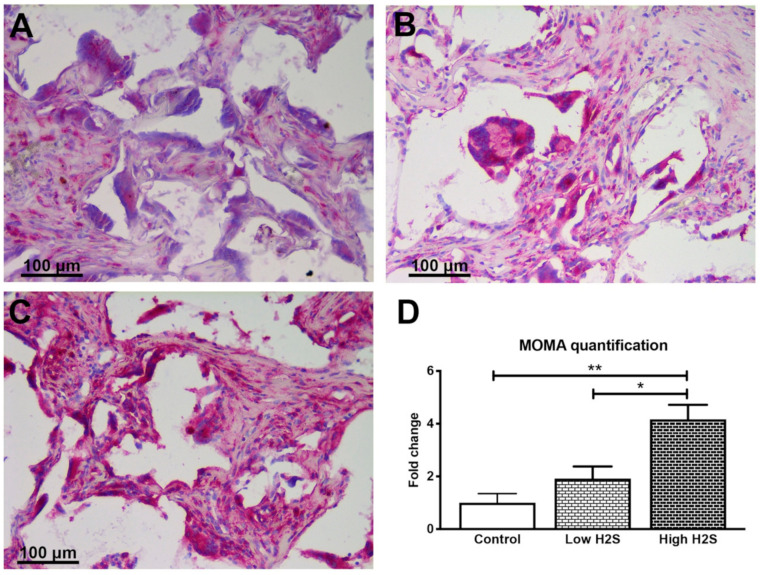
Staining of monocytes and macrophages after 63 days of implantation in nude mice. Monocytes and macrophages are stained in pink for the control group (**A**), low dose H_2_S (**B**), and high dose H_2_S (**C**). (**D**) depicts the quantification of the staining; the number of strong positive pixels per mm^2^ of scaffold was normalized to the control (*n* = 6). Mean and standard error of the mean are plotted; a statistical analysis was carried out using a one-way ANOVA with a Tukey post-hoc test, *p* < 0.05 (*), *p* < 0.001 (**).

In contrary with the results of the C57BL/6 mice, the intensity of the monocyte and macrophage staining was shown to increase in a statistically significant and dose-dependent manner (Figure 6). In the control scaffolds, monocytes and macrophages are present, but this increased twofold with the low dose H_2_S treatment (1.9 ± 0.5). The high H_2_S treatment even further increased the intensity of the monocyte and macrophage staining with a fold change of 4.2 ± 0.5. In addition, the literature states that nude mice have an exaggerated macrophage response [16]. In accordance with the literature, we observed a higher number of MOMA-2 positive pixels per mm^2^ of scaffold, 223,358 ± 71,364 and 72,532 ± 31,919, in the nude mice of the control groups compared to the C57BL/6 mice, respectively (Figure S1B).

## 4. Discussion

The formation of a stable vascular network within a subcutaneous scaffold is a complex process; it involves, among others things, angiogenic signals, migration and proliferation of endothelial cells, and formation of vascular structures. The exact role of H_2_S in the process of angiogenesis is still a subject of debate. There are only a few published in vitro studies [10,11] and even less in vivo data is available [10]. Here we investigated the role of the immune system on the angiogenic potential of H_2_S. In nude mice, which lack T cells and therefore have a deficient T-cell mediated immune response, we showed that intraperitoneal injections of a fast-releasing H_2_S donor resulted in significantly reduced vascularization of subcutaneously implanted scaffolds compared to saline-treated controls. In C57BL/6 mice with a fully functional immune system, the opposite was found; the vascularization of our subcutaneous scaffold was significantly increased after treatment with the fast-releasing H_2_S donor. This was reflected in the increased angiogenesis gene CD105 and the increased number of blood vessels at the protein level. The difference between both animal strains indicates that the T cells are involved in the H_2_S angiogenic potential. Yang and coworkers showed that H_2_S activates DNA methylation of Foxp3 T regulatory cells to promote immune tolerance [17], indicating an anti-inflammatory environment in the mice with a normal T-cell response. Differences in the immune response were also found between the immunocompetent and immunocompromised mouse model in the presence of monocytes and macrophages. This might be related to the fact that we used a setting with biomaterials that provoke a foreign body response in the first weeks after implantation [6]. Monocytes, macrophages, and foreign body giant cells play an important role in initiating the foreign body response, followed by fibroblast infiltration into the implanted scaffold. The foreign body response often occurs in the first three weeks after implantation. After that, wound healing occurs, accompanied by neovascularization of the scaffold [18]. We observed an increased presence of monocytes and macrophages in the nude mice in the H_2_S treated groups after 63 days suggesting that the foreign body response is still ongoing, while our previous study showed that this was dampened between 2 and 4 weeks [6]. H_2_S is associated with both pro- and anti-inflammatory responses [19,20,21]. During a pro-inflammatory response, it has been found that H_2_S increases the production of TNF alpha and activates monocytes [19,20]. The monocytes and macrophages are known to play an important role in tissue regeneration; for example, both these cells types influence vascular remodeling [22]. The increase in monocytes and macrophages could therefore be a factor in the observed decrease of scaffold vascularization in the nude mice. Since we observed impaired vascularization, we hypothesize that, in our study, H_2_S promotes the differentiation of pro-inflammatory M1 macrophages [23]. These M1 macrophages could induce epithelial to mesenchymal transition (EMT) [24], which might explain the reduced number of endothelial cells in nude mice. In contrary, it seems that H_2_S in C57BL/6 mice has an anti-inflammatory effect, since a reduced number of macrophages and monocytes was found. However, to confirm this hypothesis, further experiments are needed and it remains to be elucidated how H_2_S affected different lymphoid cells in order to pro-long this effect until 35 days after the last NaHS injection.

Our data supports the view that H_2_S has angiogenic potential in an immunocompetent environment and this effect seems to be concentration dependent. Here we found that the effect of H_2_S was more profound in both animal strains when using the lower dosage. Other studies have also shown that the lower dosages of H_2_S are more effective; higher H_2_S dosages seem to fail in promoting vascularization [10,11]. Cai and coworkers [10] also tested H_2_S in a biomaterial setting and showed increased neovascularization of a subcutaneous placed Matrigel plug after 7 days of intra peritoneal injections with NaHS (10–50 μmol/kg). In addition, they also found that the H_2_S effect was lost when the dose was higher than 200 μmol/kg NaHS. Several other in vivo studies in other settings without a foreign body response against a biomaterial have shown that H_2_S induces angiogenesis in highly inflamed tissues. For example, daily H_2_S treatment can selectively restore chronic ischemic tissue function [25,26] and improve ventricular remodeling after heart failure [27].

In this study, we did not distinguish between the direct and indirect effects of H_2_S. H_2_S itself has a broad range of effects depending on several factors, such as concentration, tissue type, and inflammatory status. But when H_2_S reacts with oxygen, polysulfides are formed and these can also have a broad range of physiological functions [28]. Furthermore, NaHS does not provide long-term controlled release of H_2_S. This burst release could have side effects like local cytotoxicity or limiting H_2_S effects on vascularization. To this end, clinical application would benefit from a quick noninvasive method to measure H_2_S concentration and a H_2_S donor that provides controlled release, like natural or synthetic donors [29]. However, the results might be different with a slow-releasing H_2_S donor.

## 5. Conclusions

Our findings demonstrate that the immune system plays an important role in the effect of H_2_S treatment on vascularization of subcutaneous scaffolds. In a compromised immune system, H_2_S decreases vascularization, whereas in a normal working immune system, it can stimulate vascularization. These observations suggest that H_2_S may be beneficial for the outcome of islet transplantation in a subcutaneous scaffold as treatment for type 1 diabetes, but at the same time our data warrant care in choosing correct models for studying vascularization processes as efficacy of H_2_S in immunocompromised animals was lower than in fully immunocompetent animals.

## Figures and Tables

**Table 1:** Assay IDs of RT-PCR genes.

Gene	Assay ID
CD31 (PECAM1)	Mm01242576_m1
VEGFa	Mm00437306_m1
VE-Cadherin (CDH5)	Mm00486938_m1
Angiopoietin 1	Mm00456503_m1
Angiopoietin 2	Mm00545822_m1
CD105 (Endoglin)	Mm00468252_m1
GAPDH	Mm99999915_g1

**Table 2:** Gene expression of vascularization markers after 63 days of implantation in C57BL/6 mice. Data is expressed as mean fold change ± standard error of the mean (*n* = 6). A one-way ANOVA with Tukey post-hoc test (*p* < 0.05) did not show any significant differences between the groups.

Gene	Control	Low H_2_S	High H_2_S
VEGFa	1.0 ± 0.1	0.9 ± 0.1	1.1 ± 0.01
VE-Cadherin (CDH5)	1.1 ± 0.2	1.7 ± 0.4	1.9 ± 1.0
Angiopoietin 1	1.1 ± 0.3	1.3 ± 0.4	1.6 ± 0.6
Angiopoietin 2	1.0 ± 0.1	1.1 ± 0.4	1.7 ± 0.5

**Table 3:** Gene expression of vascularization markers after 63 days of implantation in nude mice. Data are expressed as mean fold change ± standard error of the mean (*n* = 6). A one-way ANOVA with Tukey post-hoc test (*p* < 0.05) did not show any significant differences between the groups.

Gene	Control	Low H_2_S	High H_2_S
VEGFa	1.0 ± 0.1	1.0 ± 0.2	0.6 ± 0.04
VE-Cadherin (CDH5)	1.0 ± 0.1	0.9 ± 0.1	0.8 ± 0.1
Angiopoietin 1	0.9 ± 0.1	1.3 ± 0.2	1.5 ± 0.2
Angiopoietin 2	1.0 ± 0.1	0.9 ± 0.03	1.0 ± 0.2
CD105 (Endoglin)	1.0 ± 0.1	1.1 ± 0.1	1.0 ± 0.1

## Supplementary Materials

The following are available online at https://www.mdpi.com/2218-273X/10/5/722/s1, Figure S1: Number of CD31-positive blood vessels and MOMA-positive pixels per mm^2^ scaffold after 63 days. Mean and standard error of mean are plotted (*n* = 6). Statistical analysis using a Mann Whitney test (*p* < 0.05) showed significant differences (*) between the nude and C57BL/6 mice for the CD31 staining, but not for the MOMA-2 protein expression, Figure S2: Fall time during dynamic inhaled gas test. During the dynamic inhaled gas test anesthetized mice start with breathing 20% of oxygen, with a custom-made sensor the signal from the oxygen sensitive tubes in the scaffold is measured when changing from 20% to 100% of oxygen. The time until a plateau occurs at 100% was reported as the fall time, which is an indication of the vascularization status of the scaffold. Mice were treated with saline (control), low dose of H_2_S, and high dose of H_2_S during 28 days (grey area). Mean and standard error of mean are plotted (*n* = 6); statistical analysis was carried out using a two-way ANOVA with a Bonferroni post-hoc test.
