# Supplementary material for: The Effect of a Fast-Releasing Hydrogen Sulfide Donor on Vascularization of Subcutaneous Scaffolds in Immunocompetent and Immunocompromised Mice
Source: Biomolecules. 2020 May 6;10(5):722. doi: 10.3390/biom10050722 (PMC7277536; doi:10.3390/biom10050722)
Supplement: Supplementary file 1 [file biomolecules-10-00722-s001.pdf]

Supplementary Material

Differences between both Mice Strains

To investigate if there are differences in vascularization and immune response between the two mice strains, the protein expression from the control groups of both studies are compared. Comparing the raw CD31 staining counts of the control groups, demonstrated that the number of blood vessels in our subcutaneous scaffold is significantly higher in the immunocompromised nude mice compared to the immunocompetent C57BL/6 mice after 63 days of implantation (Figure S1A), respectively 40.5 ± 7.5 and 11.0 ± 1.2. Comparing the raw MOMA staining data of the control group of both mice stains, showed that the number of MOMA-2 positive pixels per mm^2 of scaffold seemed to be higher in the nude mice then in the C57BL/6 mice (Figure S1B), respectively 223358 ± 71364 and 72532 ± 31919. Although this effect is not significant, it is in accordance with literature that suggests nude mice have an exaggerated response of macrophages [14].

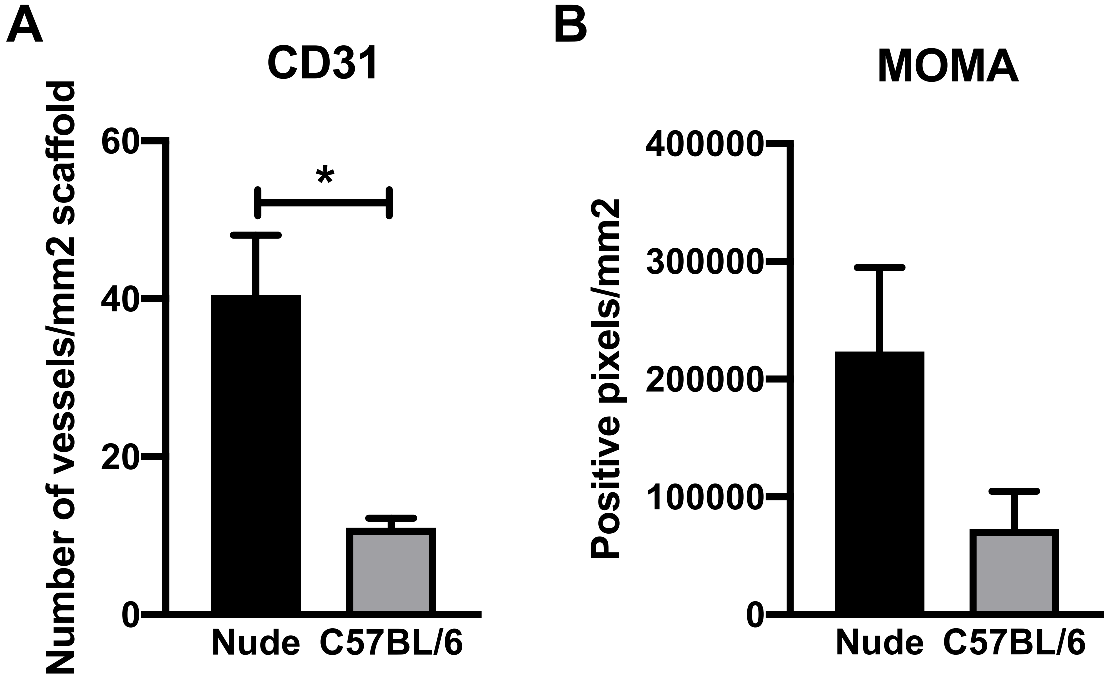

Figure S1. Number of CD31-positive blood vessels and MOMA-positive pixels per mm^2 scaffold after 63 days**.** Mean and standard error of mean are plotted (n = 6). Statistical analysis using a Mann Whitney test ($p < 0.05$) showed significant differences (*) between the nude and C57BL/6 mice for the CD31 staining, but not for the MOMA-2 protein expression.

In Vivo Oxygen Measurements

In the study with the nude, immunocompromised mice we also tested the functionality of the vasculature in vivo, but this was done using a different, non-invasive optical technique. For this, oxygen sensitive tubes were placed in the scaffold before implantation. A dynamic inhaled gas test was performed on day 3, 7, 14, 21, 35, 49, and 63 after implantation. This test provided a unique understanding of the ability of the vasculature to transport oxygen into the devices in vivo. Oxygen was changed from 20 to 100% and the time to reach the 100% oxygen in the scaffold was reported as the fall time. Expected was that the fall time would decrease over time when the vascularization of the scaffolds was improving. However, no statistically significant differences were found between the groups (Figure S2). This method required long-term anesthesia and the fur of the C57BL/6 mice would have interred with the signal, so therefore we used a different method for the C57BL/6 mice.

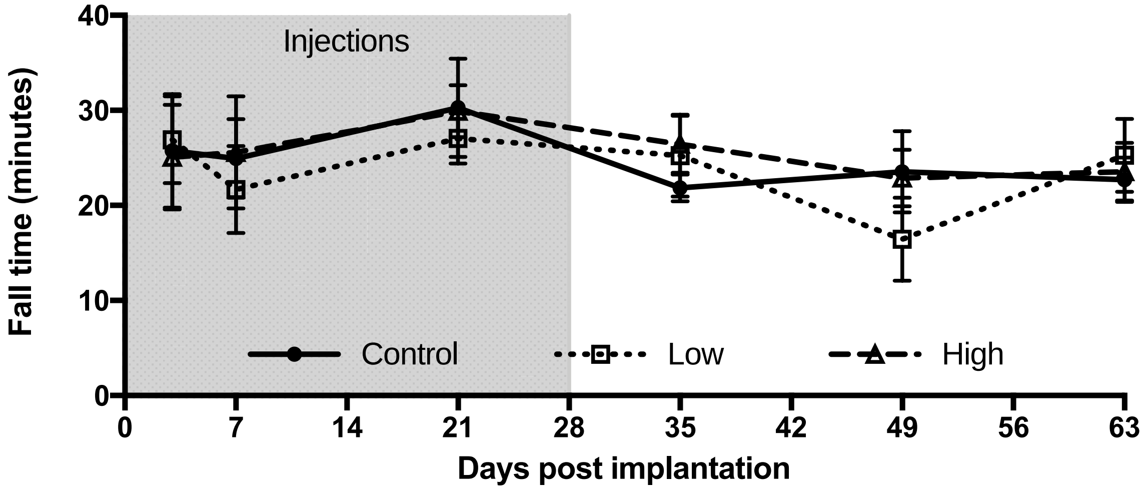

Figure S2. Fall time during dynamic inhaled gas test. During the dynamic inhaled gas test anesthetized mice start with breathing 20% of oxygen, with a custom-made sensor the signal from the oxygen sensitive tubes in the scaffold is measured when changing from 20% to 100% of oxygen. The time until a plateau occurs at 100% was reported as the fall time, which is an indication of the vascularization status of the scaffold. Mice were treated with saline (control), low dose of H_2S, and high dose of H_2S during 28 days (grey area). Mean and standard error of mean are plotted (n = 6); statistical analysis was carried out using a two-way ANOVA with a Bonferroni post-hoc test.
